# Supplementary material for: Increased contribution of parasites in microbial eukaryotic communities of different Aegean Sea coastal systems
Source: PeerJ. 2023 Dec 19;11:e16655. doi: 10.7717/peerj.16655 (PMC10740597; doi:10.7717/peerj.16655)
Supplement: Supplemental Information 2 [file peerj-11-16655-s002.pdf]

Figure S1.
Map of the sampling area

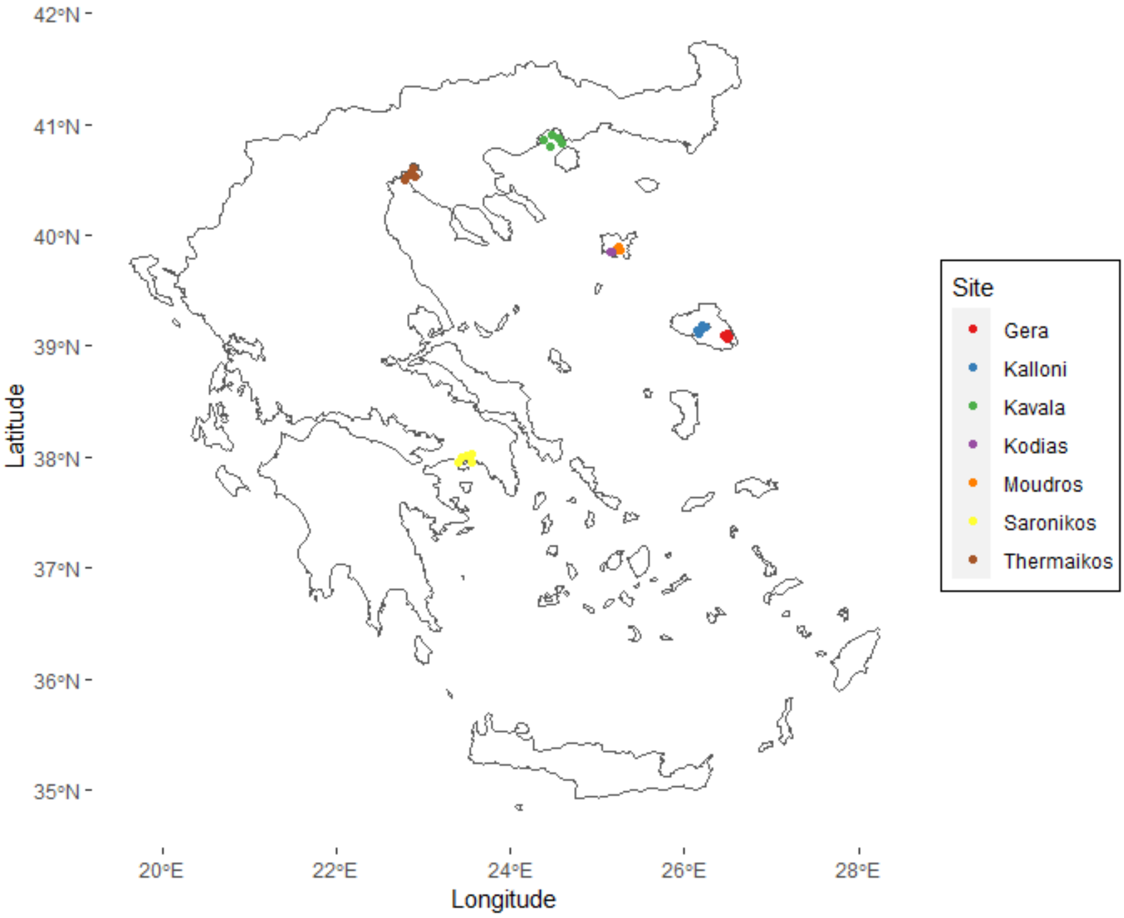

Figure S2a.
Within Gulfs Similarities calculated using Bray-curtis distances between communtities. Black lines: median value; crosses: mean values; grey line: 95% confidence interval.

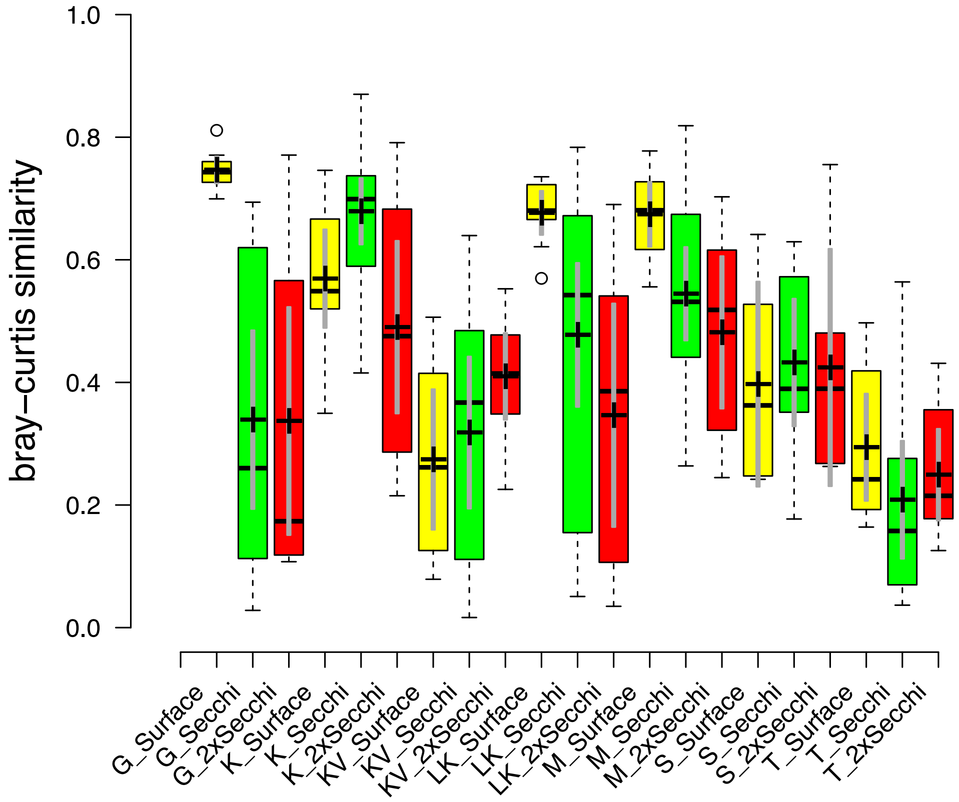

Figure S2b.
Between Gulfs Similarities calculated using Bray-curtis distances between all samples. Black lines: median value; crosses: mean values; grey line: 95% confidence interval.

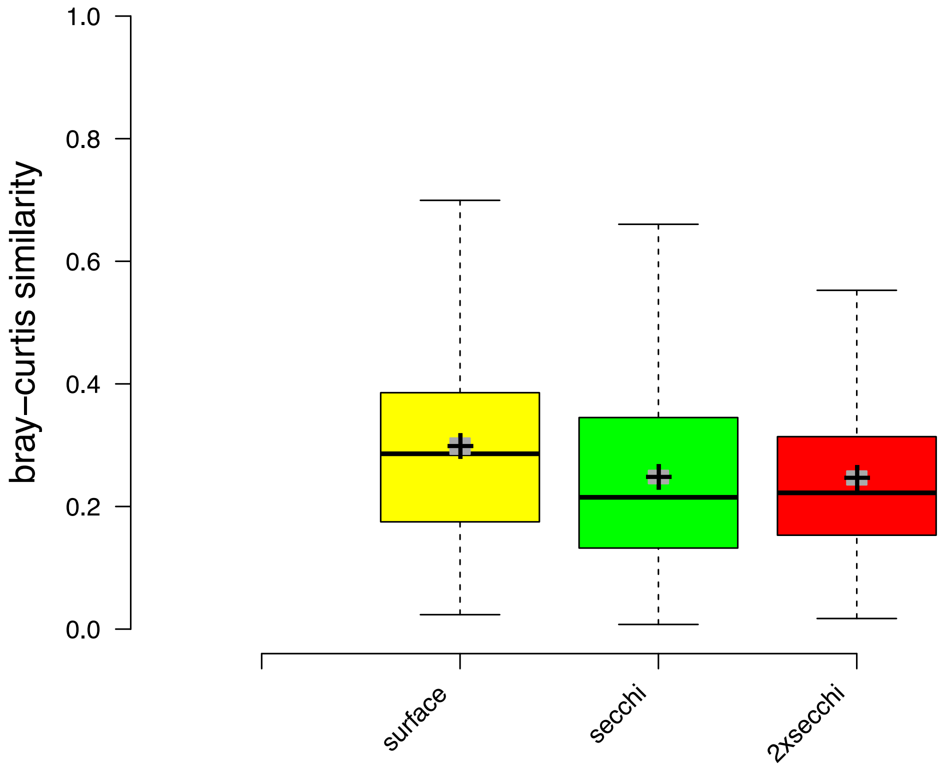

Figure S3. OTUs (a) and sequences (b) relative abundances representing top-generalists (B >15), generalists (10<B<15), specialists (1<B<10) and extreme specialists (B=1)

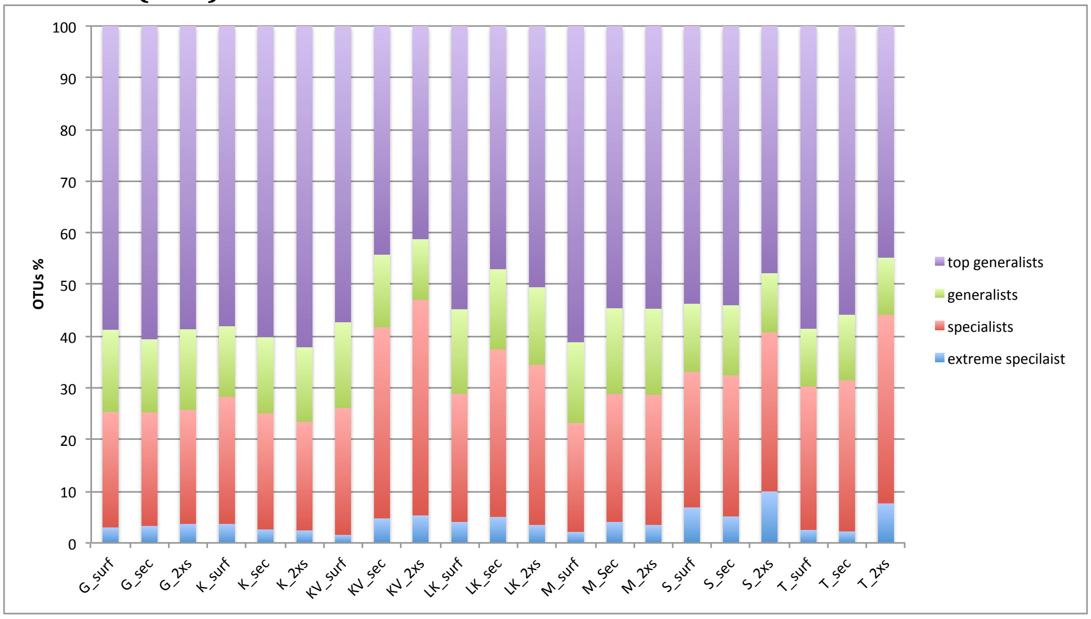

a)

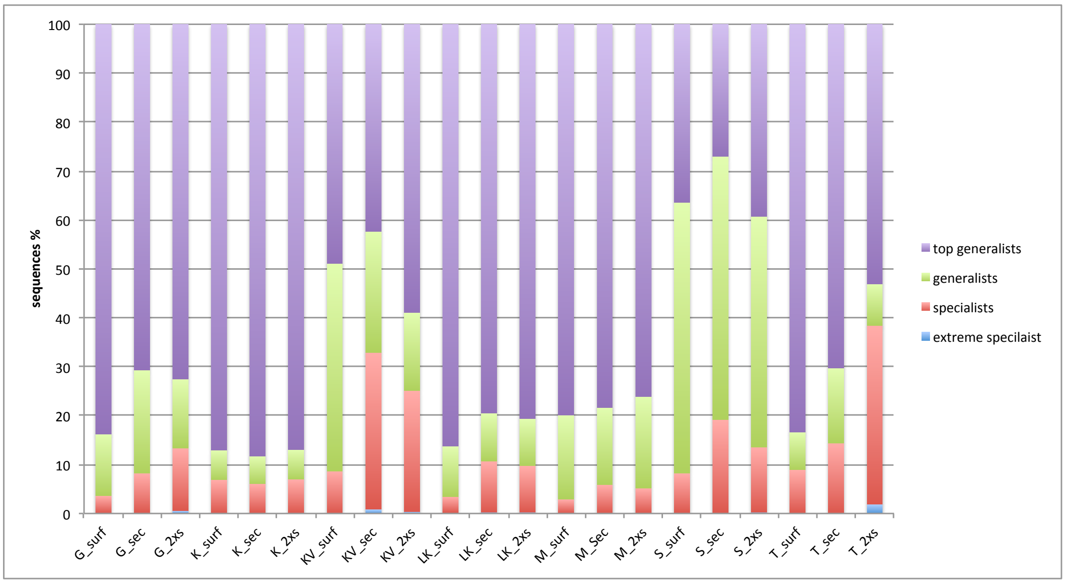

b)
